# Technological, Organizational, and Environmental Factors Influencing Social Media Adoption by Hospitals in Switzerland: Cross-Sectional Study

**DOI:** 10.2196/16995

**Published:** 2020-03-09

**Authors:** Michael Beier, Sebastian Früh

**Affiliations:** 1 University of Applied Sciences of the Grisons Chur Switzerland

**Keywords:** social media, social media adoption, hospitals, Switzerland, organizational technology adoption, TOE framework

## Abstract

**Background:**

Social media platforms are important tools for hospitals. These platforms offer many potential benefits in various areas of application for hospitals to connect and interact with their stakeholders. However, hospitals differ immensely in their social media adoption. There are studies that provide initial findings on individual factors influencing social media adoption by hospitals, but there is no comprehensive and integrated model.

**Objective:**

This study aimed to develop a comprehensive model of social media adoption by hospitals in the context of the Swiss health care system and to test the model with empirical data from Switzerland.

**Methods:**

To develop our model, we applied the general technology-organization-environment framework of organizational technology adoption and adapted it to the specific context of social media adoption by hospitals in Switzerland. To test our model, we collected empirical data on all 283 hospitals in Switzerland and identified the accounts they operate on 7 different social media platforms (Facebook, Google+, Twitter, Instagram, LinkedIn, XING, and YouTube). We tested the hypotheses of our model by means of binary logistic regression (dependent variable: platform adoption) and negative binomial regression (dependent variable: number of different platforms adopted).

**Results:**

Our general model on social media adoption received broad support. Overall, hospitals in Switzerland are more likely to adopt social media if they have a higher share of patients with voluntary health insurance or have a higher patient volume. In contrast, they are less likely to operate their own social media accounts if they are associated with a hospital network. However, some hypotheses of our model received only partial support for specific social media platforms; for instance, hospitals in Switzerland are more likely to adopt XING if they provide an educational program and are more likely to adopt LinkedIn if they are located in regions with higher competition intensity.

**Conclusions:**

Our study provides a comprehensive model of social media adoption by hospitals in Switzerland. This model shows, in detail, the factors that influence hospitals in Switzerland in their social media adoption. In addition, it provides a basic framework that might be helpful in systematically developing and testing comprehensive models of social media adoption by hospitals in other countries.

## Introduction

### Background

Social media has changed the way actors in health care interact and relate with each other in numerous ways [1]. One key actor in this field is hospitals, which have a central position in regional and national health care systems. Social media provides hospitals with many potential benefits in various areas of application but also forces them to adapt the ways in which they connect with their stakeholders. In this regard, hospitals might use social media platforms as a communication and marketing tool to reach new patients, provide information about current health topics, establish their presence in the general public, or present their service offerings [2-5]. In addition, hospitals can apply their social media channels to enhance service delivery to their patients [6-8]. Hospitals often use social media channels for educational purposes in the teaching and training of medical students and doctors and apply these channels for employer branding and recruiting [9-11]. Finally, as Web content on health care topics often is of questionable quality, hospitals’ social media channels might also act as trustworthy curators and reliable sources for online health information in the society [1,6,8].

In contrast to the outlined importance of the topic, research on social media adoption by hospitals is still in its infancy. Initial research in this field focused on the nationwide adoption rates of hospitals with regard to specific social media platforms [8,12-16]. Comparisons of the findings of these studies show remarkable differences in the hospitals’ rates of social media adoption among different countries, regions, hospital types, and social media platforms. However, deeper insights into the interplay of factors influencing social media adoption by hospitals, which could explain these differences, are scarce so far. Exploratory studies provide initial findings on individual factors influencing the adoption of social media platforms by hospitals [8,14,15]. Nevertheless, there is no comprehensive and integrated model of social media adoption by hospitals. Such a model would allow us to better understand the differences in the adoption rates described earlier. In addition, a deeper understanding of the quantitative patterns of social media adoption by hospitals would support and supplement further qualitative and quantitative research on the strategies, processes, and content of social media usage by hospitals.

In this study, we developed a comprehensive model on social media adoption by hospitals in the context of the Swiss health care system and tested the model with empirical data from Switzerland. To develop the model, we applied the general technology-organization-environment (TOE) framework of organizational technology adoption [17] and adapted it to the specific context of social media adoption by hospitals. Furthermore, we considered relevant aspects of the Swiss health care system as we assumed that some factors of influence on social media adoption by hospitals can be understood only by considering specific characteristics of the surrounding health care system [18,19].

The Swiss health care system ranks high in many indicators and is highly valued by patients. Nevertheless, it is challenged by high and rising costs and faces physician shortage [20]. In 2012, Switzerland introduced a case-based remuneration scheme (Swiss Diagnosis Related Groups, DRGs) for hospital inpatient services. In this scheme, hospitals are reimbursed a certain fee depending on the patients’ diagnoses and region (canton) of residence [19]. Before this change, most patients could only choose a hospital in their home region (canton). With the new system, patients have more freedom to choose a hospital nationwide. However, there are differences between the 2 main types of insurance in Switzerland [20,21]. The first insurance type is mandatory for everybody (Mandatory Health Insurance) and covers all general services of the health care system in accordance with the regional DRG rate. The second type is complementary and voluntary (Voluntary Health Insurance, VHI) and provides patients who pay an additional insurance premium enhanced reimbursements and advanced services, such as free choice of hospitals nationwide, single rooms with a higher level of hospital accommodation, and free choice of hospital doctors. Most hospitals in Switzerland provide services to patients with both types of health insurance. However, patients with VHI allow hospitals to generate revenues in addition to the DRG rates of their medical diagnoses.

### Research Model and Hypotheses

A hospital’s decision to officially run its own account on a specific social media platform can be seen as a specialization of organizational technology adoption. Within the general field of organizational technology adoption, Tornatzky and Fleischer [17] developed the TOE framework, which is a generic framework that comprehensively covers potential areas of relevant influence on organizational technology adoption structured by 3 different contexts: a technological context, an organizational context, and an environmental context [22]. The TOE framework has been extensively applied to study hospitals’ adoption of various information technologies [23-25]. A strength (and weakness) of the TOE framework is its general applicability [22]. On the one hand, it can be adapted to all technologies used by organizations. On the other hand, its high level of abstraction requires sufficient adjustments and specifications to allow meaningful application to a specific technology in a specific industry.

Therefore, in this study, we developed an integrated research model on social media adoption by hospitals by following the TOE approach. We developed hypotheses on the factors influencing social media adoption with regard to the specific technological, organizational, and environmental contexts of hospitals. For this purpose, we systematically adapted the fundamental concepts of the TOE approach to social media as the technology to be adopted and to the specific context of hospitals. At certain points, we also relied on specific characteristics of the Swiss hospital system.

#### Technological Context

The technological context of the TOE framework addresses questions on the benefits and costs related to the adoption of a new technology [26,27]. The perceived benefits of new information technology can comprise assumed possibilities for the generation of a relative competitive advantage by its application [24,28] or enhanced processes because of internal improvements or better collaboration with external partners [29,30]. In contrast, perceived costs mainly arise from expected integration costs and barriers in accordance with existing internal or external technologies in use [26,27]. Therefore, a lack of relevant competencies or resources leads to higher perceived costs and barriers [31,32].

With regard to the outlined calculus of organizational technology adoption, social media platforms are a unique technology. From a technological cost perspective, social media appears rather simple as the underlying third-party platforms are readily available at no cost and are user friendly [33]. Therefore, the technological affordances of social media platforms for organizations and their employees may be perceived as low compared with those of more complex and expensive information systems. However, significant integration costs and barriers are mainly based on the internal processes of communication, marketing, and compliance, which must be adapted to the affordances of social media communication. For hospitals, the potential benefits of social media adoption primarily lie in the enhanced interactions and relationships with relevant stakeholders [34,35]. Correspondingly, questions of internal compatibility and capacities for social media adoption by hospitals are mainly strategic and are operational questions regarding marketing and communication [36]. Therefore, hospitals might decide to run their own accounts on social media platforms based on their expectations of the extent to which doing so will improve the effectiveness or efficiency of relevant stakeholder interactions and relationships [37-39].

Patients with additional VHI represent one external stakeholder group that is very important for many hospitals (in Switzerland) and is accessible via social media platforms. This group is very attractive for hospitals as health insurance compensates hospitals beyond the DRG rate for advanced accommodation and other amenities [20]. Patients with VHI have more options in their hospital choice and are more strongly influenced by the nonmedical characteristics of hospitals, eg, ambience, accommodation, and comfort [40,41]. Similar to the hotel sector, these characteristics are particularly suitable for an effective and efficient presentation in social media [42,43]. Correspondingly, we hypothesized that the more important patients with VHI are for a hospital, the higher is its propensity to adopt social media. We thus proposed the following hypothesis:

H1a: The higher the share of patients with VHI in hospitals, the more likely they are to adopt social media.

The education and training sector is another important field in which hospitals might seek to improve their interactions and relations with their stakeholders via social media [14]. Social media is particularly suited for health care organizations to relate and interact with communities of medical students and practitioners [9,44]. Social media platforms provide effective channels for hospitals to communicate with potential, actual, and former participants of their educational programs [45]. Therefore, it can be expected that hospitals that provide such programs are more likely to run their own social media accounts. In alignment with this assumption, findings from studies in the United States and China show that hospitals that are involved in graduate medical education or affiliated with a university are more likely to adopt social media [8,14,15]. Thus, we proposed the following hypothesis:

H1b: Hospitals that provide an educational program are more likely to adopt social media.

#### Organizational Context

The organizational context of the TOE framework refers to the organizations’ internal structures and processes that may facilitate or constrain the adoption of new information technology [18,46]. One basic organizational factor included in many TOE studies on the adoption of technology in hospitals is the hospital size [23,24,47,48]. The findings of these studies are mainly similar, showing that larger hospitals adopt new technologies faster. Similarly, initial exploratory findings in the United States show that larger hospitals (measured by the number of beds) are more likely to adopt social media than smaller ones [14,15]. However, in organizational technology adoption, hospital size covers two different arguments. On the one hand, hospital size is seen as an indicator of the extent of internal infrastructure. In this regard, a certain size means that a hospital has all necessary resources and capabilities as well as the ability to set up additional assets to apply new technology adequately [23,24,48]. On the other hand, hospital size is seen as an indicator for the operational volume of patients served by the hospital [47,49,50]. In these cases, some technologies can be applied effectively or efficiently only if a hospital has a certain volume of patients. The adoption of social media platforms does not necessitate any internal infrastructure for hospitals but mainly shows benefits of enhanced effectiveness or efficiency depending on the size of the respective stakeholder groups with which a hospital wants to connect [51-53]. Therefore, regarding social media adoption by hospitals, the organization size might be more a matter of operational patient volume than of infrastructure capacities. Consequently, we proposed the following hypothesis:

H2a: The higher the patient volume in hospitals, the more likely they are to adopt social media.

Another organizational factor of technology adoption refers to internal structures [54]. One important aspect of hospital structure in this context is centralization [18,49]. Early studies argue that technology adoption is easier with decentralized structures when the technology is only locally applied within a hospital (for instance, in a specific department). In contrast, adoption decisions for technologies that affect a whole hospital benefit from centralized decision-making rights [50]. As social media adoption raises questions of ownership within an organization, centralized decision making should be beneficial for social media adoption decisions [55]. Accordingly, exploratory findings from outside the hospital sector show that centralized leadership fosters social media adoption in organizations as it makes resource allocations and decision-making processes easier [56]. In Switzerland, there is a tendency to form consolidated hospital organizations that run several hospital sites [20]. Currently, approximately 30% of the hospitals in Switzerland run at more than one site [57]. For a hospital running at several sites, each with its own directorates, it might be more difficult or take longer to reach an agreement on the adoption of social media. On the one hand, more individual interests of different parties and differences in local needs must be considered [18]. On the other hand, more decision makers have the opportunity to block a decision on social media adoption because of risk concerns [33]. Overall, we hypothesized that a more complex and dispersed structure of a hospital organization is related to a lower propensity for social media adoption. Thus, we proposed the following hypothesis:

H2b: The more sites hospitals run, the less likely they are to adopt social media.

#### Environmental Context

The environmental context of the TOE framework refers to all factors outside an organization that may facilitate or constrain the adoption of new information technology [22]. One important factor in this context is external partners and their influence on the organizations’ decision making regarding technology adoption [37]. In this regard, the hospitals’ decision to adopt new information technology can be influenced by their association with a parent group or hospital network (eg, Hirslanden or the Swiss Medical Network in Switzerland). Such groups are seen as effective communicators and facilitators of innovations for their member hospitals, and they often provide technological support and shared services to implement new technologies [23]. Similarly, exploratory studies have shown that hospitals that are affiliated with a health or hospital system are more likely to be present on social media [14,15]. However, these exploratory studies do not explicitly focus on the organizational social media adoption by individual hospitals. Thus, they do not explicitly differentiate between cases where a hospital runs its own social media account on a platform versus cases where a hospital is only co-represented in the social media account of a parent group, hospital network, or health system. With regard to organizational social media adoption, however, this difference is significant. Correspondingly, regarding individual hospitals and their social media adoption, we hypothesized a negative influence of their affiliation to a hospital network. On the one hand, in some cases, parent networks run social media accounts as shared services representing their member hospitals with social media accounts of the whole network [58]. Therefore, for a focal hospital, the perceived benefits of running its own account (in addition to the network account) on a social media platform should be diminished compared with the situation of an independent hospital. On the other hand, some hospital networks might apply a centralized communication strategy that does not allow member hospitals to run their own accounts in specific social media platforms. Both patterns reduce the propensity of an individual hospital to run its own social media accounts if it is affiliated with such a network. Thus, we proposed the following hypothesis:

H3a: Hospitals that are affiliated with a hospital network are less likely to adopt social media.

Another important environmental factor of technology adoption is competition intensity [59]. Several studies on information technology adoption by hospitals have observed a positive relationship between the level of competition intensity that hospitals face and their propensity to adopt new information technology [47,60,61]. On the one hand, in an environment with high competition intensity, organizations try to change the rules of the competition by adopting new technology and try to decrease the threat of competitors by leveraging new ways to outperform their rivals [31,62]. On the other hand, under higher competition intensity, managers perceive higher peer pressure to adopt new technologies [18,63]. Initial research on general social media adoption proposes a similar relationship between the competition intensity and social media adoption of organizations [64]. Thus, we proposed the following hypothesis:

H3b: The higher the competition intensity that hospitals face, the more likely they are to adopt social media.

In our research model, we analyzed social media adoption at the organizational level of hospitals. However, in the model, we also covered two other organizational levels as attributes of these hospitals. On the one hand, we incorporated lower-level units of these hospitals by analyzing the number of sites they run (H2b) as an attribute of their internal structure (organizational context). On the other hand, we included higher-level entities by analyzing if the hospitals are affiliated with a hospital network or parent group (H3a) as an attribute of their external partner relationships (environmental context).

**Figure 1:**
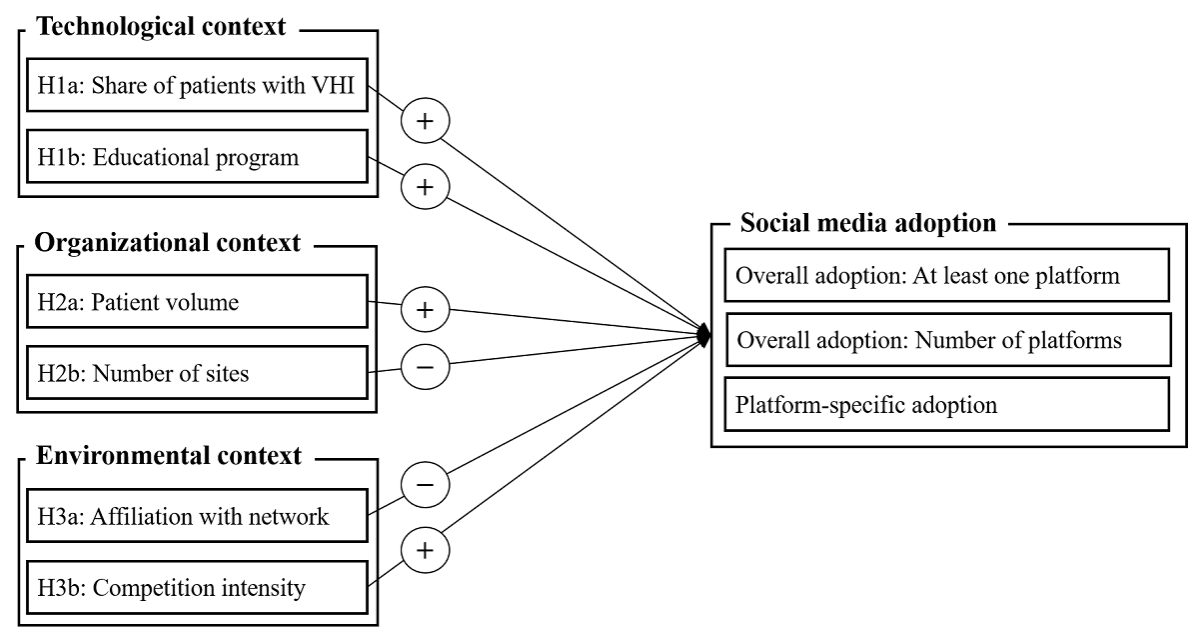
The research model. VHI = voluntary health insurance. The + sign indicates a hypothesized positive relation. The − sign indicates a hypothesized negative relation.

Our research model covered social media adoption as the dependent variable of our hypotheses in 3 different ways: first, the binary overall adoption of at least one social media platform; second, the total number of different social media platforms adopted, covering the breadth of overall adoption; and third, the binary adoption of specific social media platforms. Figure 1 presents an overview of our integrated research model.

## Methods

### Data Collection

To test our research model, we used data on all Swiss hospitals from several different sources. We collected data on the overall population of hospitals in Switzerland. To achieve this, we applied the official list of key figures for all Swiss hospitals from the Federal Office of Public Health (FOPH) in Switzerland [57]. The list provided us with all relevant data on the hospitals’ characteristics needed for our study. Additional data on the main languages in different regions (cantons) of Switzerland were gathered from the Federal Statistical Office (FSO) of Switzerland [65]. Finally, we used the FOPH list to collect data on the social media presence of all hospitals in Switzerland on different social media platforms.

In line with previous studies [14-16], we collected the social media data for all hospitals in 3 steps. In the first step, we performed a Google search for the official homepages of all hospitals on the FOPH list. During this step, we had to reduce our sample from 283 hospitals on the FOPH list to 279 hospitals. Two of the hospitals on the list had closed. Another hospital organization is listed as 2 suborganizations on the FOPH list. As it was not possible to merge the data of these 2 suborganizations with the social media data, we had to exclude these 2 entries from our sample. In the second step, we gathered all social media accounts that were linked from the official homepages of hospitals in our sample. On the basis of these links, we identified the relevant social media platforms for this study. We included all social media platforms where at least 10 hospitals in our sample provided a link on their official homepage to their own social media account on the platform. Correspondingly, in further data collection, we included 7 platforms: Facebook (126 links from individual hospital homepages to their accounts on the platform), YouTube (89 links), LinkedIn (62 links), Twitter (53 links), XING (36 links), Instagram (25 links), and Google+ (15 links). In the third step, we completed our systematic search for social media accounts of the hospitals in our sample. To achieve this, we performed a Google search for the name of each hospital in combination with the name of each platform. In addition, we searched each social media platform for the accounts of each hospital. To exclude informal social media accounts operated under the name of hospitals, we applied a low-threshold minimal criterion. Therefore, in our data collection, we did not include unofficial Facebook pages (pages for public places automatically generated by the platform itself) or accounts on the other platforms where no background picture or profile picture was uploaded and no *About Us* text was provided. The final social media data were gathered from September 17 to 19, 2018. We collected only publicly available data from the social media channels of hospitals. No persons were directly involved. The dataset generated and analyzed in this study is available from the corresponding author on reasonable request.

### Measures

With regard to the independent variables of our research model, we measured the hospitals’ share of patients with VHI by the percentage of patients with VHI among all hospital patients, operationalized as a value between 0 and 100. We used a dummy variable to indicate whether a hospital ran an educational program. This variable took the value of 1 if a hospital was listed on the FOPH list as a provider of educational programs for medical students or doctors and 0 if not. We measured the patient volume of hospitals in values of 1000 patients per year. The number of sites a hospital ran was operationalized as a hospital’s number of locations listed in the FOPH list. We operationalized the hospitals’ affiliation to a network with a dummy variable. This variable took the value of 1 for all hospitals whose online communication included any indication that the hospital was associated with a parent group or hospital network (shared homepage, any shared social media channel, hyperlink to the parent group from any online channel, or listed as a member on the group website). In all cases without such an indication, the dummy variable took the value of 0. As competition in the Swiss hospital market is mainly focused within regions (cantons) [20], competition intensity was considered as the number of hospitals on the FOPH list that were active in the same region (canton).

We operationalized social media adoption as the dependent variable in our research model in 3 different ways. First, we used 7 dummy variables (one for each social media platform) to measure platform-specific social media adoption by hospitals. For each platform, the dummy variable took the value of 1 if a hospital operated its own account on the platform (not via a hospital group as a representative of the hospital) and 0 if this was not the case. Second, we operationalized the binary overall adoption of social media by hospitals with an additional dummy variable if a hospital had a social media presence in at least one of the platforms included in our study. Third, we measured the total number of different platforms wherein a hospital operated its own accounts as the sum of the dummy variables of the specific platforms with a numerical variable.

We applied additional measures to control for further influences on the hospitals’ social media adoption. Prior results indicate that the ownership of hospitals (especially privately vs publicly owned hospitals) might influence their social media adoption [14,15]. Therefore, we controlled for public hospitals with a dummy variable that took the value of 1 if a hospital was listed with the legal status of a public company in the FOPH list and 0 in all other cases. As Switzerland is a multilingual country with different language regions (German, French, and Italian as main languages in specific cantons), we also used dummy variables to control for possible language-related effects. With one dummy variable each, we indicated whether a hospital was located in a canton where the main language was German, French, or Italian, as classified by the FSO of Switzerland [65].

### Analytical Approach

As described earlier, the dependent variables of our research model are operationalized on the basis of 8 binary dummy variables (7 dummies for specific platforms and 1 for any platform) and 1 numeric variable (the total number of different platforms). Correspondingly, for each binary dependent variable, we computed a separate binary logistic regression model. The total number of different social media platforms used by a hospital shows the characteristics of count data. Furthermore, we found significant evidence of overdispersion in the data [66]. Therefore, we tested our hypotheses with regard to the total number of different social media platforms as the dependent variable by means of a negative binomial regression. All analyses were performed in R (The R Foundation for Statistical Computing, Vienna, Austria). Details are provided in Multimedia Appendix 1.

We also tested for multicollinearity issues by computing correlations (Kendall tau) between all explanatory variables in our analyses. As all correlations were less than 0.45, no multicollinearity issues were indicated regarding our regression analyses [67].

## Results

### Descriptive Results

In Table 1, we present the respective adoption rates of all binary dependent variables of our research model. First, we display the overall social media adoption rate of hospitals using at least one social media platform (Any). In the other rows, we present the adoption rates for the specific social media platforms included in our data collection. The data show that 74.6% (208/279) of all hospitals in Switzerland run their own social media accounts on at least one platform. The adoption rates of specific platforms range from a maximum of 58.4% (163/279) (Facebook) to a minimum of 13.3% (37/279) (Instagram).

**Table 1:** Rates of social media adoption by Swiss hospitals (N=279).

Platform	Adoption rate, n (%)
Any	208 (74.6)
Facebook	163 (58.4)
Google+	79 (28.3)
Twitter	57 (20.4)
Instagram	37 (13.3)
LinkedIn	114 (40.9)
XING	57 (20.4)
YouTube	98 (35.1)

Furthermore, 59.5% (166/279) of the hospitals in our sample run an educational program, 23.3% (65/279) of them are affiliated with a hospital network, and 20.4% (57/279) are public hospitals. Most hospitals in our sample are located in a German-speaking region (207/279, 74.2%), 21.1% (59/279) are located in a French-speaking region, and 4.7% (13/279) are located in an Italian-speaking region.

For all other nonbinary variables, we provide descriptive statistics in Table 2. The hospitals’ share of patients with additional VHI range between 0% and 100%, with a mean of 25.7%. The patient volumes per year for the hospitals in our sample show a maximum of 55,200 patients and a mean of 4940 patients. Hospitals run at a maximum of 22 sites, with a mean of 2.01. Competition intensity varies from no competition to 42 hospitals in the same region (canton). The total number of different social media platforms used by a hospital (dependent variable) ranges from 0 to 7, with a mean of 2.17.

**Table 2:** Descriptive results of nonbinary variables (N=279).

Variable	Mean (SD)	Median	Minimum value	Maximum value
H1a: Share of patients with voluntary health insurance (%)	25.70 (26.400)	19.22	0.00	100.00
H2a: Patient volume (in 1000)	4.94 (8.445)	1.56	0.01	55.20
H2b: Number of sites	2.01 (2.663)	1.00	1.00	22.00
H3b: Competition intensity	20.22 (12.160)	16.00	1.00	42.00
Dependent variable: Number of platforms	2.17 (1.935)	2.00	0.00	7.00

### Regression Results

We have presented our regression results in 9 models (results of models 1 to 3 are provided in Table 3, results of models 4 to 6 in Table 4, and results of models 7 to 9 in Table 5). Model 1 covers the logistic regression results of the binary overall adoption of at least one social media platform (any platform) by the hospitals. Model 2 displays the negative binomial regression results for the total number of different social media platforms adopted by hospitals (number of platforms). Models 3 to 9 show a binary logistic regression model of platform-specific social media adoption for each individual social media platform.

The results for the binary logistic regression models (all models except model 2) are presented as odds ratios (ORs). The ORs indicate the expected changes in the hospitals’ odds of social media adoption when the respective explanatory variable changes by one unit. Correspondingly, we have displayed the results for the negative binomial regression in model 2 (on the number of different platforms adopted) as incidence rate ratios (IRRs). Similar to the ORs, the IRRs indicate the expected changes in the number of different social media platforms that the hospitals adopt when the respective explanatory variable changes by one unit. For all regression models, we displayed the 95% confidence intervals of the ORs or IRRs and *P* values for each explanatory variable as well as the Nagelkerke *R^2^* for the overall regression model. For the categorical data on language regions, we applied German-speaking regions as a reference category.

**Table 3:** Regression results of models 1 to 3.

Variable	Model 1: Any platform^a^			Model 2: Number of platforms^b^			Model 3: Facebook^c^	
	OR^d^ (95% CI)	*P* value	IRR^e^ (95% CI)		*P* value	OR (95% CI)		*P* value
H1a: Share of patients with voluntary health insurance	1.01 (1.00^f^-1.03)	.02	1.01 (1.00^f^-1.01)		<.001	1.01 (1.00^f^-1.02)		.03
H1b: Educational program	1.37 (0.72-2.60)	.33	1.13 (0.90-1.40)		.30	0.95 (0.54-1.67)		.85
H2a: Patient volume	1.08 (1.01-1.15)	.02	1.03 (1.02-1.04)		<.001	1.06 (1.01-1.11)		.009
H2b: Number of sites	1.00 (0.89-1.13)	.97	0.96 (0.92-1.00)		.06	0.87 (0.77-0.99)		.03
H3a: Affiliation with network	0.28 (0.14-0.53)	<.001	0.47 (0.36-0.61)		<.001	0.54 (0.30-0.98)		.04
H3b: Competition intensity	1.00 (0.97-1.02)	.80	1.00 (1.00-1.01)		.48	1.00 (0.98-1.03)		.76
Public hospital	0.88 (0.38-2.06)	.77	1.25 (0.97-1.61)		.09	1.38 (0.66-2.90)		.39
French-speaking region	0.65 (0.32-1.34)	.25	0.81 (0.63-1.04)		.09	0.66 (0.35-1.26)		.21
Italian-speaking region	0.36 (0.10-1.30)	.12	0.66 (0.40-1.08)		.09	0.95 (0.29-3.15)		.93
Constant	2.31 (1.06-5.03)	.04	1.59 (1.21-2.10)		.001	1.20 (0.60-2.41)		.61

^a^Nagelkerke *R^2^*=0.151.

^b^Nagelkerke *R^2^*=0.248.

^c^Nagelkerke *R^2^*=0.112.

^d^OR: odds ratio.

^e^IRR: incidence rate ratio.

^f^The value is greater than 1 but rounds to 1.00.

**Table 4:** Regression results of models 4 to 6.

Variable	Model 4: Google+^a^		Model 5: Twitter^b^		Model 6: Instagram^c^	
	OR^d^ (95% CI)	*P* value	OR (95% CI)	*P* value	OR (95% CI)	*P* value
H1a: Share of patients with voluntary health insurance	1.02 (1.00^e^-1.03)	.01	1.02 (1.01-1.04)	.002	1.02 (1.01-1.04)	.002
H1b: Educational program	0.85 (0.45-1.61)	.61	1.16 (0.51-2.67)	.72	0.74 (0.29-1.89)	.53
H2a: Patient volume	1.04 (1.00^e^-1.08)	.03	1.11 (1.05-1.16)	<.001	1.08 (1.03-1.13)	<.001
H2b: Number of sites	0.96 (0.85-1.08)	.46	0.85 (0.68-1.06)	.14	0.83 (0.60-1.14)	.25
H3a: Affiliation with network	0.50 (0.25-1.03)	.06	0.07 (0.02-0.28)	<.001	0.21 (0.07-0.70)	.01
H3b: Competition intensity	1.00 (0.98-1.03)	.84	0.99 (0.96-1.01)	.30	0.99 (0.96-1.02)	.63
Public hospital	2.06 (0.96-4.42)	.06	1.97 (0.81-4.76)	.13	1.46 (0.50-4.28)	.49
French-speaking region	0.34 (0.15-0.78)	.01	0.70 (0.28-1.74)	.44	0.82 (0.30-2.19)	.69
Italian-speaking region	0.57 (0.14-2.34)	.44	0.38 (0.06-2.31)	.29	0.00 (0.00-∞)	.99
Constant	0.30 (0.14-0.66)	.003	0.18 (0.07-0.50)	<.001	0.12 (0.04-0.37)	<.001

^a^Nagelkerke *R^2^*=0.125.

^b^Nagelkerke *R^2^*=0.305.

^c^Nagelkerke *R^2^*=0.225.

^d^OR: odds ratio.

^e^The value is greater than 1 but rounds to 1.00.

**Table 5:** Regression results of models 7 to 9.

Variable	Model 7: LinkedIn^a^			Model 8: XING^b^		Model 9: YouTube^c^	
	OR^d^ (95% CI)	*P* value	OR (95% CI)		*P* value	OR (95% CI)	*P* value
H1a: Share of patients with voluntary health insurance	1.01 (1.00^e^-1.02)	.03	1.01 (0.99-1.03)		.26	1.01 (1.00^e^-1.02)	.04
H1b: Educational program	1.41 (0.77-2.58)	.26	2.69 (1.11-6.53)		.03	1.16 (0.60-2.28)	.66
H2a: Patient volume	1.06 (1.02-1.10)	.004	1.10 (1.04-1.16)		<.001	1.10 (1.05-1.15)	<.001
H2b: Number of sites	0.94 (0.84-1.05)	.27	0.96 (0.85-1.08)		.48	0.97 (0.87-1.08)	.55
H3a: Affiliation with network	0.44 (0.23-0.85)	.01	0.13 (0.04-0.45)		.001	0.05 (0.01-0.16)	<.001
H3b: Competition intensity	1.03 (1.01-1.05)	.01	1.01 (0.98-1.04)		.44	1.00 (0.98-1.02)	.95
Public hospital	1.23 (0.59-2.57)	.58	1.76 (0.73-4.25)		.21	1.50 (0.67-3.34)	.32
French-speaking region	2.48 (1.28-4.80)	.007	0.02 (0.00-0.25)		.003	0.44 (0.20-0.99)	.05
Italian-speaking region	0.75 (0.20-2.73)	.66	0.00 (0.00-∞)		.98	0.51 (0.13-2.09)	.35
Constant	0.19 (0.09-0.41)	<.001	0.09 (0.03-0.27)		<.001	0.43 (0.19-0.95)	.04

^a^Nagelkerke *R^2^*=0.173.

^b^Nagelkerke *R^2^*=0.405.

^c^Nagelkerke *R^2^*=0.316.

^d^OR: odds ratio.

^e^The value is greater than 1 but rounds to 1.00.

#### Technological Context

Hypotheses H1a and H1b of our research model addressed the technological context of social media adoption by hospitals. The general proposition of our model in this context was that hospitals are more likely to run their own account on a social media platform when they have higher expectations that this platform provides benefits for their organizational communication with stakeholder groups that are individually important for them. In this regard, hypothesis H1a suggested that hospitals with a higher share of patients with VHI are more likely to adopt social media. We found broad support for hypothesis H1a in our regression results that showed significant positive effects of the hospitals’ share of patients with VHI on their social media adoption. More specifically, we found significant positive effects for the adoption of at least one social media platform (model 1), the total number of different platforms adopted (model 2), and the platform-specific adoption of all platforms included in our study except the online business-related social network, XING (model 8). This result seemed plausible as XING is a regional business platform focused on German-speaking countries [68] and may be less helpful for relationship marketing and community building with patients. In contrast to the result for XING, the regression result for hospitals’ adoption of LinkedIn (the other business platform included in our study) showed a significant positive effect of their share of patients with VHI (model 7). This difference might be explained by 2 factors. First, LinkedIn is a global business platform that facilitates connections to wealthy and internationally oriented patients all over the world, whereas XING is mainly focused only on German-speaking countries. Second, hospitals might use LinkedIn as an indirect way to attract patients. Instead of reaching patients directly via social media, hospitals might aim to reach national and international physicians and other health experts in their role as referring doctors or influencers in the field [69,70]. In Switzerland, patients do not need a referral from their attending physician to choose a hospital in all cases [20], but these physicians still have an important influence on the patients’ hospital choices [70,71].

Another important sector in which hospitals can establish and maintain beneficial relationships with external parties via social media is the education and training sector [14]. Correspondingly, hypothesis H1b suggested that hospitals providing an educational program are more likely to adopt social media. We found only partial support for hypothesis H1b as our regression results showed only a significant positive effect of the hospitals’ provision of an educational program on their propensity to run an own XING account (OR 2.69, 95% CI 1.11-6.53; *P*=.03). However, this result seems convincing, as one main focus of XING is the market for coaching and training [72]. In contrast, reaching business-related stakeholders in a private context on general purpose social media platforms might seem less beneficial for hospitals. Similar patterns have already been observed regarding social media applications in business-to-business marketing [73].

Overall, the results support the general perspective we developed in our research model regarding the technological context of hospitals’ social media adoption. The main drivers in this context are the potential benefits that hospitals can derive from their social media presence. However, the concrete benefits that hospitals perceive as relevant are influenced by their individual market situation and positioning strategies. Therefore, hospitals with a stronger market focus on patients with VHI show higher adoption propensities on social media platforms for private individuals, physicians, and health professionals. In contrast, only hospitals offering education or training on the market show higher propensities to adopt a business-related social network that specializes in the education and training market.

#### Organizational Context

Hypotheses H2a and H2b of our research model addressed the organizational context of the hospitals’ social media adoption. Hypothesis H2a stated that hospitals with a higher patient volume are more likely to run their own social media accounts. This hypothesis was fully supported by the results of all regression models we have tested. For hospitals serving more patients, additional efforts for social media communication are more cost-efficient as the costs per patient decrease with higher patient numbers. Such hospitals also tend to have more resources for communication and marketing activities. Furthermore, hospitals with more patients might also attract greater public attention and therefore be under more institutional pressure to have a social media presence [74,75].

Another aspect of the organizational context of technology adoption is the centralization and distribution of decision rights. In this regard, hypothesis H2b claimed that hospitals that have more locations and therefore have more local directorates show lower propensities for social media adoption. This hypothesis received only partial support. The results of our regression models showed only a significant negative effect of the number of hospital sites on the hospitals’ propensity to run their own Facebook account (OR 0.87, 95% CI 0.77-0.99; *P*=.03). Facebook is by far the most widely used online social network among individuals in Switzerland [76] and is well known for facilitating negative word of mouth [77]. In recent years, Facebook has been involved in many scandals on data misuse and privacy breaches [78,79]. Therefore, local directorates of hospital sites might have higher risk aversion and greater fear of a potential loss of control with regard to the communication of internal and external stakeholders on this platform [80]. Correspondingly, local directorates of hospital sites may show higher propensities to veto an organization-wide adoption of Facebook. Hospitals might also be less likely to operate a Facebook account when they run more sites as they do not want to provide a public space for employees and other stakeholders to communicate on issues related to the hospital, especially among different sites [81].

#### Environmental Context

Hypotheses H3a and H3b of our research model covered the environmental context of social media adoption by hospitals. In this regard, hypothesis H3a proposed that hospitals that are associated with a hospital network are less likely to have their own social media accounts. We found broad support for hypothesis H3a in our regression results, which showed significant negative effects of the hospitals’ affiliation with a network on their social media adoption. More specifically, we found significant negative effects for the adoption of at least one social media platform (model 1), the total number of different platforms adopted (model 2), and the platform-specific adoption of all platforms included in our study except Google+ (model 4). Our results extend previous research by explaining in more detail how affiliation with a group of hospitals influences the social media adoption of individual hospitals. Previous research showed that hospitals that are affiliated with a health or hospital system are more likely to be represented on social media by their own social media account or the account of their affiliated group [14,15]. Our study showed an opposite effect as we only considered the social media presence of accounts run by a focal hospital itself. As a concretization of previous research, we found strong support for our hypothesis that group affiliations reduce hospitals’ propensity to run their own social media accounts. Indeed, affiliation with a hospital network might increase hospitals’ propensity to be present on social media in some way (be it directly with their own account or indirectly via a network account). However, such an affiliation also decreases their propensity to run accounts on social media by themselves. On the one hand, social media accounts run by a hospital network might already provide sufficient benefits to their member hospitals that some of them perceive that there is no need to run individual accounts in addition. On the other hand, in cases where a hospital network applies a centralized communication strategy, member hospitals might also be encouraged by the network not to run individual accounts in addition to the collective accounts of the network. As described earlier, to address research questions on organizational social media adoption, it is essential to include only social media accounts run by a focal organization as the dependent variable. If not, by also including accounts run by an associated network, the analysis levels of an organization and its environment get mixed up.

Regional competition intensity is another aspect of the environmental context of the hospitals’ social media adoption. In this regard, hypothesis H3b suggested that hospitals located in regions with higher competition intensity are more likely to run their own social media accounts. This hypothesis was supported only by the results of our binary logistic regression with regard to LinkedIn accounts (OR 1.03, 95% CI 1.01-1.05; *P*=.01). On the one hand, the explanation for this finding may be similar to that for the hospitals’ orientation toward patients with VHI. Correspondingly, hospitals under more competitive pressure might not address patients directly via social media. Instead, they may establish and maintain contacts with physicians and other health experts (as referring doctors or influencers in the field) via the business-related social network, LinkedIn [69]. On the other hand, as Switzerland faces significant physician shortage, another plausible explanation for the observed pattern is that higher competition intensity among hospitals might be less a question of patients and more a question of the regional labor market for physicians [20]. In both cases, LinkedIn would be the most appropriate platform in our sample.

## Discussion

### Principal Findings

Previous studies provide initial empirical evidence of individual factors influencing hospitals’ adoption of social media [8,14,15]. However, a comprehensive and integrated model of the factors influencing the adoption of social media by hospitals was missing. To our knowledge, this study is the first to provide such a model. To develop our model, we applied the general TOE framework [17] and adapted it systematically to the context of our study. We derived specific hypotheses on technological, organizational, and environmental factors influencing the adoption of social media platforms by hospitals. We tested our hypotheses with regard to the hospitals’ overall adoption (adoption of at least one platform and the total number of platforms adopted) and platform-specific adoption with regard to the 7 social media platforms most commonly used by hospitals in Switzerland. Table 6 displays our final model with an overview of the empirical evidence we found in our data.

**Table 6:** Final model and empirical evidence.

Explanatory variables			Overall adoption				Platform-specific adoption													Hypothesis support	
			At least one platform		Number of platforms		Facebook		Google+		Twitter		Instagram		LinkedIn		XING		YouTube		
**Technological context**																					
	H1a: Share of patients with voluntary health insurance	+^a^		+		+		+		+		+		+		NS^b^		+		Broad	
	H1b: Educational program	NS		NS		NS		NS		NS		NS		NS		+		NS		Partial	
**Organizational context**																					
	H2a: Patient volume	+		+		+		+		+		+		+		+		+		Full	
	H2b: Number of sites	NS		NS		−^c^		NS		NS		NS		NS		NS		NS		Partial	
**Environmental context**																					
	H3a: Affiliation with network	−		−		−		NS		−		−		−		−		−		Broad	
	H3b: Competition intensity	NS		NS		NS		NS		NS		NS		+		NS		NS		Partial	

^a^The + sign indicates a significant positive relation (*P*<.05).

^b^NS: no significant relation (*P*>.05).

^c^The − sign indicates a significant negative relation (*P*<.05).

Our overall research model received comprehensive support. All hypotheses received at least some empirical support in the data analyses, as expected. However, our findings also allowed us to derive more detailed patterns of the platform-specific adoption of social media by hospitals. In particular, our findings regarding the technological context indicated that social media platforms should be perceived not as homogeneous technology but as a heterogeneous set of specific tools for different communication purposes with different stakeholder groups. In this regard, our results showed, for instance, that hospitals with a stronger market focus on patients with VHI tend to adopt social media platforms for private individuals (eg, Facebook, Twitter, Instagram, or YouTube), physicians, and health professionals (LinkedIn). In contrast, hospitals offering education or training on the market showed higher propensities to adopt a business-related social network that specializes in the education and training market (XING). Overall, this finding indicates that hospitals choose the social media platforms on which they should be present based on their relevant stakeholder groups. Our findings also show that hospitals with higher patient volumes generally tend to adopt social media more, independent of specific social media platforms. Finally, we found broad support for our hypothesis that individual hospitals affiliated with a hospital network or group are less likely to adopt any social media.

### Limitations and Opportunities for Future Research

Our study has 3 main limitations that also indicate opportunities for future research. The first limitation is that we applied only abstract data on hospital characteristics and binary data on social media adoption. Therefore, we observed quantitative patterns of social media adoption but were not able to directly address the logic really applied during the underlying adoption decisions and adoption processes. Further (especially qualitative) research is necessary to understand in more detail how hospitals make adoption decisions and actually adopt social media. To achieve this, future studies could apply interview data of key informants who are directly involved in decision-making and implementation processes to gather more concrete information on the considerations, structures, and processes leading to social media adoption by hospitals.

The second limitation of this study is the low explanatory power of some of the regression models. Although our overall model is supported comprehensively by the data, some of the regression models have only low model strength (eg, model 3 on Facebook adoption with a Nagelkerke *R^2^* of 0.112), indicating that in these models our explanatory variables can only explain a small part of the variance of the dependent variable. Therefore, future research might search for further factors influencing the adoption of social media by hospitals and add them to our model. However, to this end, it might be useful (or necessary) to develop specific models for individual social media platforms.

The third limitation of this study is its limited generalizability as we focused on Switzerland, a country with a rather unique hospital sector. Indeed, it is also a strength of this study that we adapted the model to the specific context of the Swiss health care system as this allowed us to develop more concrete and context-specific hypotheses. However, this national specialization of the model also reduces the direct generalizability of our findings. Therefore, future research should adapt the model to the relevant characteristics and empirically test it in other national health care systems. In this regard, our model, as a context-specific adaptation of the general TOE approach, allows future studies to integrate further hypotheses adapted to other specific national contexts. For instance, in the technical context of our model, we identified patients with additional VHI and participants of educational programs as relevant stakeholder groups that Swiss hospitals can reach effectively and efficiently via social media platforms. For studies in other countries, other relevant stakeholder groups (eg, private or charitable fundraising, recruiting, and societal or political legitimacy) could be integrated into the model. Furthermore, the organizational and environmental contexts of the model can be adapted to specific characteristics of the hospitals and their relevant settings in other countries. In this regard, our model provides a starting point as a basic framework for the further development of more comprehensive and detailed models on the social media adoption of hospitals in all countries.

## Appendix

Multimedia Appendix 1 Specifications R statistics.

## Abbreviations

DRG: diagnosis-related group
FOPH: Federal Office of Public Health
FSO: Federal Statistical Office
IRR: incidence rate ratio
OR: odds ratio
TOE: technology-organization-environment
VHI: voluntary health insurance
