# Supplementary material for: Technological, Organizational, and Environmental Factors Influencing Social Media Adoption by Hospitals in Switzerland: Cross-Sectional Study
Source: J Med Internet Res. 2020 Mar 9;22(3):e16995. doi: 10.2196/16995 (PMC7351265; doi:10.2196/16995)
Supplement: Multimedia Appendix 1 [file jmir_v22i3e16995_app1.pdf]

Appendix 1: Specifications R Statistics

All analyses were performed in R:
R version 3.6.1 (2019-07-05) -- "Action of the Toes"
Copyright (C) 2019 The R Foundation for Statistical Computing
https://www.r-project.org
[accessed 2019-11-08].

Specific applications:

1. Binary logistic regression:
   glm function

2. Test for overdispersion in count data:
   AER package (Version 1.2-8): dispersiontest function
   https://cran.r-project.org/web/packages/AER/index.html
   [accessed 2019-11-08].

3. Negativ binomial regression:
   MASS package (Version 7.3-51.4): glm.nb function
   https://cran.r-project.org/web/packages/MASS/index.html
   [accessed 2019-11-08].

4. Calculation of Nagelkerke R^2 for all regression models:
   rcompanion package (Version 2.3.7): nagelkerke function
   https://cran.r-project.org/web/packages/rcompanion/index.html
   [accessed 2019-11-09].
